# Diagnostic Imaging of the Chest

**Published:** 1987-05

**Authors:** David Mackenzie-Crooks


**DIAGNOSTIC IMAGING OF THE CHEST**

**by Paul Goddard MD FRCR.**
**Publishers: Churchill Livingstone**

This is a short, readable, and refreshingly new approach to the radiological management of chest diseases. Not intended as a text for interpretation of basic chest radiographs, it takes the next important step by providing a welcome guide to the correct sequence of further investigation for diagnostic chest problems.

The text contains many useful lists, and is supplemented by a series of well illustrated case histories that give a step by step guide to diagnosis. The illustrations are excellent quality. All current imaging modalities are covered, and particular stress is placed on the valuable role of CT scanning in chest disease.

The book, which will sell at around £45, will give valuable advice to respiratory physicians, MRCP candidates and trainee radiologists. More experienced radiologists will benefit from reading the case histories, which can also be used for a self quiz. A useful addition to the radiological management of chest disease.

David Mackenzie-Crooks

